# Prognostic Value of Coronary Dominance in Patients Undergoing Elective Coronary Artery Bypass Surgery

**DOI:** 10.21470/1678-9741-2019-0079

**Published:** 2020

**Authors:** Emre Selcuk, Deniz Cevirme, Onursal Bugra

**Affiliations:** 1 Department of Cardiovascular Surgery, Mus State Hospital, Muş, Turkey.; 2 Department of Cardiovascular Surgery, Kartal Kosuyolu Research and Education Hospital, Istanbul, Turkey.; 3 Department of Cardiovascular Surgery, Medical Faculty, Balikesir University, Balikesir, Turkey.

**Keywords:** Patient Readmission, Confidence Intervals, Coronary Artery Bypass, Postoperative Complications, Risk, Elective Surgical Procedures

## Abstract

**Objective:**

To evaluate the clinical impact of coronary dominance type in terms of early and long-term outcomes in patients undergoing elective coronary artery bypass grafting (CABG).

**Methods:**

A total of 844 consecutive patients who underwent elective CABG were divided into two groups based on preoperative angiographic views as left dominant (LD) and right dominant or co-dominant (RD+CD). The measured outcomes were postoperative complications, 30-day mortality, long-term mortality, and major adverse cardiac and cerebrovascular events (MACCE).

**Results:**

RD+CD was present in 87.9% (n=742) and LD in 12.1% (n=102) of patients. Postoperative complications, 30-day mortality, and 30-day readmissions were similar in both groups. The median duration of follow-up was 3.4 years. LD was not an independent predictor of mortality (adjusted hazard ratio [HR] 1.53, 95% confidence interval [CI] 0.89-2.45, *P*=0.12), but it was an independent predictor of MACCE in the long term (adjusted HR 2.18, 95% CI 1.39-3.42, *P*=0.001).

**Conclusion:**

In patients undergoing elective surgical revascularization, left coronary dominance is associated with increased MACCE risk in the long term. Therefore, the assessment of coronary dominance type should be an integral part of outpatient management after CABG.

**Table:** 

Abbreviations, acronyms & symbols				
ACE	= Angiotensin-converting enzyme		ISR	= In-stent restenosis
ACS	= Acute coronary syndrome		LAD	= Left anterior descending artery
BMI	= Body mass index		LCOS	= Low cardiac output syndrome
CABG	= Coronary artery bypass grafting		LD	= Left dominance (or dominant)
CAD	= Coronary artery disease		LMCA	= Left main coronary artery
CD	= Co-dominance (or co-dominant)		LV	= Left ventricular
CI	= Confidence intervals		MACCE	= Major adverse cardiac and cerebrovascular events
COPD	= Chronic obstructive pulmonary disease		MI	= Myocardial infarction
CPB	= Cardiopulmonary bypass		PCI	= Percutaneous coronary intervention
Cx	= Circumflex artery		PDA	= Posterior descending artery
eGFR	= Estimated glomerular filtration rate		RCA	= Right coronary artery
EuroSCORE	= European System for Cardiac Operative Risk Evaluation		RD	= Right dominance (or dominant)
HR	= Hazard ratios		SD	= Standard deviation
IQR	= Interquartile range		SPSS	= Statistical Package for the Social Sciences

## INTRODUCTION

Type of coronary dominance is defined by the vessel that gives rise to the posterior descending artery (PDA), which supplies the myocardium from the inferior part of the interventricular septum. Right dominance (RD) is the most common anatomic variant, in which PDA originates from the right coronary artery (RCA). In the left dominance (LD), PDA is a branch of the circumflex artery (Cx). If the inferior part of the interventricular septum is supplied by both Cx and RCA, this variant is termed as co-dominance (CD) or balanced pattern^[1,2]^.

All three types of coronary dominance are considered to be variants of normal circulation. However, the differences between coronary dominance types may lead to different clinical outcomes in patients with coronary artery stenosis according to various studies^[3]^. Left coronary dominance has been shown to be a poor prognostic factor in patients with acute coronary syndrome (ACS) and percutaneous interventions^[4,5]^. In contrast, the knowledge about the clinical effects of the type of coronary dominance is scarce in patients undergoing surgical revascularization. Our main purpose in this study is to evaluate the prognostic value of coronary dominance differences in terms of early and long-term outcomes in patients undergoing elective coronary artery bypass grafting (CABG).

## METHODS

### Study Population and Data Source

This multi-surgeon single-center retrospective study included all patients who underwent elective CABG after coronary angiography between January 2015 and December 2015 at the Kartal Kosuyolu Research and Education Hospital in Istanbul, Turkey. The exclusion criteria were (1) emergency and urgent operations, (2) off-pump CABG, (3) previous cardiac surgery, and (4) concomitant cardiac procedures. Patients were divided into two groups (LD and RD+CD) based on coronary angiographic images using standard angiographic projections in accordance with the American College of Cardiology/American Heart Association College. RD and CD patients were in the same group because they had been previously reported with similar anatomical and clinical features^[4,5]^. Moreover, it was not possible to precisely distinguish between CD and RD patients due to diffuse coronary lesions. Angiographic images and hospital records were completely available for in-hospital outcomes. The overall survival results of all patients were obtained from the national database. The patients who were followed up outside the referenced hospital were contacted by telephone and the assessment was performed based on their medical records. The Institutional Ethical Committee of Kartal Kosuyolu Research and Education Hospital approved the study protocol. The study conformed to the principles of the Declaration of Helsinki. All the authors contributed equally to the study and were working at the abovementioned hospital during the study period.

### Procedural Characteristics

The decision for surgical revascularization was made by the consensus of at least one cardiac surgeon and a cardiologist. Surgical revascularization was performed with conventional on-pump CABG technique (internal mammary artery for left anterior descending artery and saphenous vein for other vessels). The intermittent antegrade blood cardioplegia was used routinely in all patients. Additional retrograde cardioplegia was used optionally according to the surgeon's preference. Complete revascularization was the main goal in all patients. All primary operators had experience of at least five years. All patients were discharged after CABG with single antiplatelet therapy (acetylsalicylic acid or clopidogrel), beta blocker, and statin therapy. Patients who required dual antiplatelet therapy or anticoagulation for any reason were excluded from the study to minimize the effect of differences in secondary prevention on follow-up outcomes.

### Analysis of Outcomes

In this study, early postoperative and follow-up results of the two groups (LD and RD+CD) were compared. Early outcomes included CABG-associated in-hospital complications, first 30-day mortality, and unplanned hospital readmissions. The overall follow-up information of the patients was evaluated until December 2018. Long-term endpoints of the study were determined as (1) death and (2) the composite outcome of major adverse clinical events. Major adverse cardiac and cerebrovascular events (MACCE) were defined as all-cause mortality, non-fatal myocardial infarction (MI), non-fatal stroke, and late revascularization.

All data related to mortality and the first 30-day results could be completely collected from electronic records. MACCE data was available in 96.4% of patients. Pattern of missing values was totally random (Little's test of missing completely at random, *P*=0.87)^[6]^. For this reason, performing complete-case analyses was evaluated to be appropriate for avoiding potential bias originated from missing values^[7]^. MACCE analysis was performed after listwise deletion of the patients with any missing value.

### Statistical Analysis

Categorical variables were presented as counts and frequencies; continuous variables as mean ± standard deviation or median (interquartile range [IQR]), as appropriate. Chi-square test or Fisher’s exact test was used for comparison between categorical variables. Student *t*-test or Mann-Whitney U test was used to compare continuous variables. Prognostic value of coronary dominance for all-cause mortality and MACCE was analysed with Cox proportional-hazards model. First, the univariate Cox regression model was established with coronary dominance, age, chronic obstructive pulmonary disease (COPD), chronic kidney disease (estimated glomerular filtration rate [eGFR] < 60 mL/min), and well-defined cardiovascular risk factors, such as male gender, obesity (body mass index ≥ 30 kg/m^2^), extracardiac arteriopathy, recent MI (last three months), severe left ventricular (LV) dysfunction (ejection fraction ≤ 30%), diabetes mellitus, hyperlipidemia, incomplete revascularization, and severity of coronary artery disease (anatomical SYNTAX score). Likewise, every variable was included in the multivariate analyses. Hazard ratios (HR) with 95% confidence intervals (CI) were reported. Cumulative survival and event-free survival curves were shown with Kaplan-Meier graphs. A two-tailed *P*-value of 0.05 was considered statistically significant. The Statistical Package for the Social Sciences (SPSS) software (SPSS, Chicago, Illinois, USA), version 23.0, was used for all statistical analyses.

## RESULTS

### Baseline Characteristics

A total of 844 consecutive patients who underwent elective surgical revascularization were assessed. Patients were stratified according to coronary dominance pattern (RD+CD and LD). RD+CD was present in 87.9% (n=742) and LD in 12.1% (n=102) of the study population. Patients in both groups had similar demographic characteristics and overall risk profile. There was no statistically significant difference between the two groups in terms of European System for Cardiac Operative Risk Evaluation (EuroSCORE) II levels (RD+CD 1.99% [0.50-13.6], LD 1.96% [0.50-11.25], *P*=0.08). The distribution of critical coronary lesions was similar, except for the right system stenosis, because it was more frequent in the RD+CD group. Anatomical SYNTAX scores of both groups were similar. Surgical technique and myocardial protection strategies were also similar in the two groups. In the RD+CD group, graft number, cardiopulmonary bypass time, and cross-clamp time were significantly longer than in the LD patients. Preoperative findings and procedural characteristics stratified by coronary dominance are shown in Table 1.

**Table 1:** Baseline characteristics of study population.

		Total	RD + CD	LD	*P*-value
Demographics	Patients, n (%)	844 (100)	742 ( 87.9)	102 (12.1)	
	Age (years), median (IQR)	61 (16)	61 (17)	62 (15)	0.07
	BMI (kg/m^2^), median (IQR)	28.2 (5.3)	28.3 (5.2)	27.9 (4.8)	0.25
	Male gender, n (%)	629 (74.5)	558 (75.2)	71 (69.6)	0.15
Cardiovascular risk factors	Severe LV dysfunction, n (%)	151 (17.9)	132 (17.8)	19 (18.6)	0.83
	Diabetes mellitus, n (%)	244 (28.9)	209 (28.2)	35 (34.3)	0.19
	Hypertension, n (%)	413 (48.9)	365 (49.2)	48 (47.1)	0.68
	Hyperlipidemia, n (%)	454 (41.9)	318 (42.9)	36 (35.3)	0.14
	Current smoking, n (%)	497 (58.9)	313 (42.2)	34 (33.3)	0.09
Comorbidities	COPD, n (%)	127 (15.0)	108 (14.6)	19 (18.6)	0.28
	Extracardiac arteriopathy, n (%)	151 (17.9)	134 (18.1)	17 (16.7)	0.73
	eGFR < 60 ml/min, n (%)	83 (9.8)	71 (9.6)	12 (11.8)	0.48
History	Recent MI, n (%)	316 (37.4)	276 (37.2)	40 (39.2)	0.69
	Prior PCI, n (%)	161 (19.1)	144 (19.4)	17 (16.7)	0.7
	ISR, n (%)	93 (11.0)	84 (11.3)	9 (8.8)	0.45
Culprit vessels	LMCA, n (%)	182 (21.6)	159 (21.4)	23 (22.5)	0.79
	LAD, n (%)	807 (95.6)	707 (95.3)	100 (98)	0.20
	Cx, n (%)	609 (72.2)	532 (71.7)	77 (75.5)	0.42
	Diagonal, n (%)	313 (37.1)	279 (37.6)	34 (33.3)	0.40
	Right system, n (%)	555 (65.8)	518 (69.8)	37 (36.3)	< 0.001
Severity of CAD	SYNTAX score, median (IQR)	31(8)	31 (8)	30 (8)	0.95
	Low (≤ 22), n (%)	133(15.8)	120(16.2)	13 (12.7)	
	Intermediate (23-32), n (%)	433(51.3)	377 (50.8)	56 (54.9)	
	High (≥ 33), n (%)	278( 32.9)	245 (33)	33 (32.9)	
Procedural characteristics	Graft number, median (IQR)	3 (1)	3 (1)	2 (1)	< 0.001
	CPB time (min), median (IQR)	88 (37)	90 (37)	82 (41)	0.02
	Cross-clamp time (min), mean ± SD	50 (21)	50 (24)	37 (20)	< 0.001
Myocardial protection, n (%)	Antegrade cardioplegia	547 (64.8)	483 (65.1)	64 (62.7)	
	Antegrade + retrograde cardioplegia	297 (35.1)	259 (34.9)	38 (37.3)	
	Incomplete revascularization	68 (8.1)	59 (8.0)	9 (8.8)	0.76
Discharge medication	Acetylsalicylic acid	764(90.5)	676 (91.1)	88 (86.3)	0.11
	Clopidogrel	80 (9.5)	66 (8.9)	14(13.7)	0.11
	Beta blocker	812 (96.2)	714 (96.2)	98 (96.1)	0.94
	ACE inhibitor	305 (36.1)	31 (30.4)	274 (36.9)	0.19

ACE=angiotensin-converting enzyme; BMI=body mass index; CAD=coronary artery disease; COPD=chronic obstructive pulmonary disease; CPB=cardiopulmonary bypass; Cx=circumflex artery; eGFR=estimated glomerular filtration rate; IQR=interquartile range; ISR=in-stent restenosis; LAD=left anterior descending artery; LD=left dominant; LMCA=left main coronary artery; LV=left ventricular; MI=myocardial infarction; PCI=percutaneous coronary intervention; RD+CD=right dominant or co-dominant; SD=standard deviation

### Postoperative Outcomes

Intraoperative mortality was not observed in the study group. First 30-day lethal outcome ratio was 1.9% (n=16). LD group had higher first 30-day mortality than RD+CD group but there were no statistically significant differences between both groups (RD+CD 1.8%, LD 2.9%, *P*=0.42). There was still no significant difference in first 30-day mortality after adjustment for EuroSCORE II of patients (*P*=0.51, HR_adjusted_ 1.52, 95% CI 0.42-5.4). In-hospital complications and first 30-day readmission ratios were similar in both groups (Table 2).

**Table 2:** Postoperative complications and first 30-day outcomes of study patients.

	Total	RD + CD	LD	*P*-value
Arrhythmia, n (%)	102 (12.1)	62 ( 8.4)	6 (5.9)	0.39
Infection, n (%)	61 (7.2)	50 (6.7)	11 (10.8)	0.13
Renal failure, n (%)	58 (6.9)	52 (7.0.)	6 (5.9)	0.67
Postoperative MI, n (%)	48 (5.7)	43 (5.8)	5 (4.9)	0.71
Reexploration, n (%)	45 (5.3)	40 (5.4)	5 (4.9)	0.83
Prolonged intubation (> 24 hours), n (%)	60 (7.1)	53 (7.1)	7 (6.9)	0.91
Neurologic, n (%)	20 (2.4)	16 (2.2)	4 (3.9)	0.27
LCOS, n (%)	28 (3.3)	24 (3.2)	4 (3.9)	0.71
Hospital days, median (IQR)	7 (3)	7 (3)	8 (4)	0.06
Intensive care days, median (IQR)	2 (3)	2 (3)	2 (2)	0.24
First 30-day readmission, n (%)	76 (9.0)	67 (9.0)	12 (8.8)	0.94
First 30-day mortality, n (%)	16 (1.9)	13 (1.8)	3 (2.9)	0.42

IQR=interquartile range; LCOS=low cardiac output syndrome; LD=left dominant; MI=myocardial infarction; RD+CD=right dominant or co-dominant

### Survival Outcomes and Predictive Factors of All-cause Mortality

**Fig. 1:**
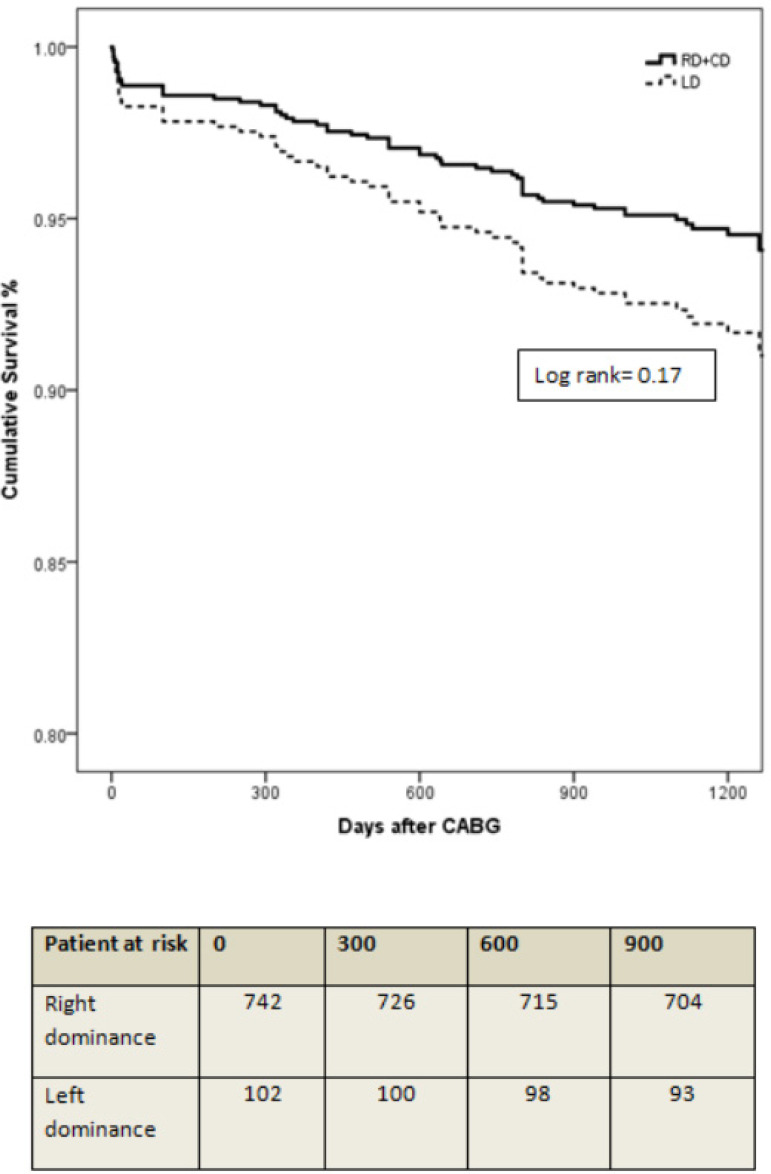
Kaplan-Meier plot for all-cause mortality. CABG=coronary artery bypass grafting; LD=left dominance; RD+CD= right dominance or co-dominance

Median follow-up period after operation was 3.4 (IQR 0.63) years. Total all-cause mortality rate was 7.2% (n=61) (RD+CD 6.7%, n=50; LD 10.8%, n=11). In the study population, type of coronary dominance was not an independent predictor of all-cause mortality according to univariate and multivariate Cox regression analyses. Age, vascular disease, and eGFR < 60 mL/min were independent predictors of all-cause mortality. The independent predictors of mortality are displayed in Table 3. Event-free survival curve from all-cause mortality during follow-up stratified by two coronary dominance types is shown in Figure 1.

**Table 3:** Independent predictors of all-cause mortality according to univariate and multivariate Cox analyses.

Variables	Univariate		Multivariate	
	HR (95% CI)	*P*-value	HR _adjusted_ (95% CI)	*P*-value
Left dominance	1.56 (0.92-3.0)	0.08	1.53 (0.89- 2.45)	0.12
Age	1.10 (1.07-1.13)	<0.001	1.35 (1.14- 1.56)	<0.001
Male gender	0.80 (0.43-1.48)	0.49	1.24 (0.72-2.15)	0.72
Obesity	1.00 (0.96-1.04)	0.96	0.91 (0.71-1.24)	0.82
Vascular disease	2.78 (1.62-4.76)	<0.001	2.04 (1.29- 3.06)	0.02
COPD	1.64 (0.91-2.97)	0.09	1.45 (0.92- 2.51)	0.09
Recent MI	1.68 (0.61-4.65)	0.31	1.42 ( 0.97-2.24)	0.31
Severe LV dysfunction	1.02 (0.51-2.00)	0.97	1.2 (0.61-1.82)	0.21
Diabetes mellitus	1.44 (0.84-2.47)	0.17	1.62 (1.01-2.03)	0.09
Hyperlipidemia	1.35 (0.94-3.40)	0.51	1.49 (0.91-2.033)	0.24
eGFR < 60 ml/min	4.11 (2.34-7.31)	<0.001	3.41 (1.178-4.21)	<0.001
Incomplete revascularization	1.42 (0.50-3.95)	0.50	1.31 (0.43-2.84)	0.12
SYNTAX score	1.4 (0.92-2.05)	0.21	1.03 (0.89-1.41)	0.11

CI=confidence interval; COPD=chronic obstructive pulmonary disease; eGFR=estimated glomerular filtration rate; HR=hazard ratio; LV=left ventricular; MI=myocardial infarction Omnibus test for multivariate Cox model resulted in *P*<0.001.

### Relationship Between Coronary Dominance and Adverse Events

**Fig. 2:**
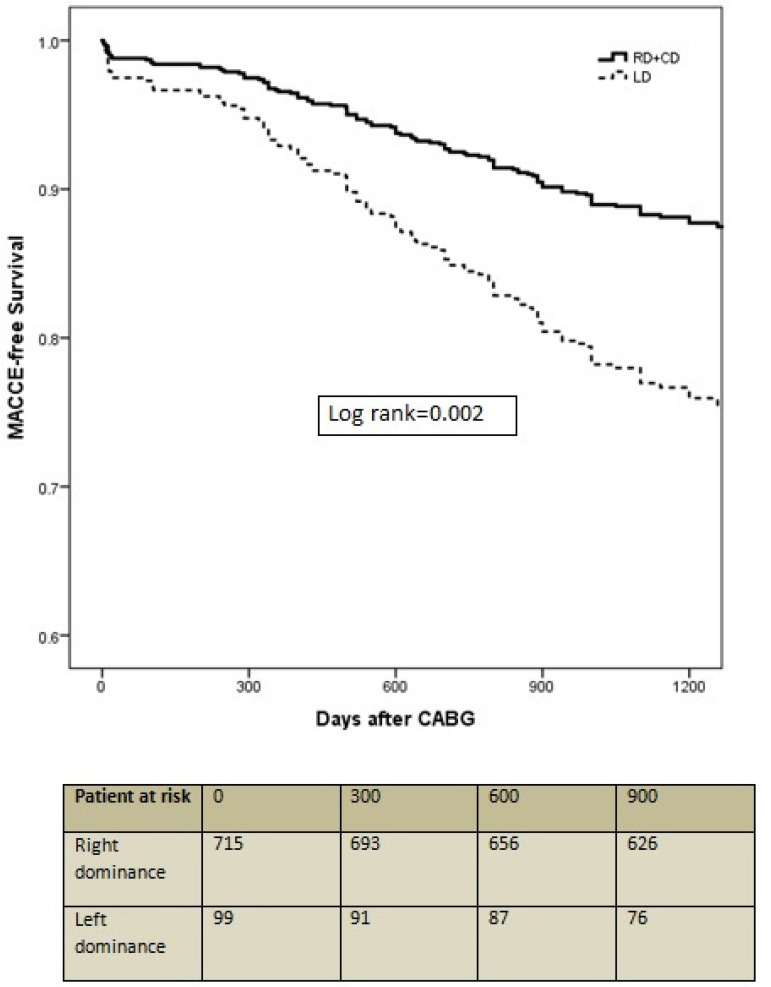
Kaplan-Meier plot for MACCE-free survival. CABG=coronary artery bypass grafting; LD=left dominance; MACCE=major adverse cardiac and cerebrovascular events; RD+CD= right dominance or co-dominance

A total of 130 (16%) patients had MACCE during the follow-up period (Table 4). MACCE were much more pronounced in LD patients than in the RD+CD group (RD+CD 14.5%, n=104/715; LD 26.3%, n=26/99; *P*=0.005). LD, age, vascular disease, COPD, severe LV dysfunction, and eGFR < 60 ml/min were univariate predictors of MACCE in the study patients. LD, vascular disease, severe LV dysfunction, and eGFR < 60 ml/min showed strong association with MACCE in multivariate analysis (Table 5). MACCE-free survival curves of the two types of coronary dominance diverge further as the follow-up period increases (Figure 2).

**Table 4:** Clinical outcomes in patients with left and non-left coronary dominance.

	Total* (n=814)	RD + CD (n=715)	LD (n=99)	*P*-value
Non-fatal stroke	30 (3.7)	27 (3.8)	3 (3.0)	0.71
Non-fatal MI, n (%)	58 (7.1)	45 (6.3)	13 (13.1)	0.01
Late revascularization, n (%)	46 (5.7)	37 (5.2)	9 (9.1)	0.11
All-cause mortality, n (%)	61 (7.2)	49 (6.9)	11 (11.1)	0.25
MACCE, n (%)	130 (16.0)	104 (14.5)	26 (26.3)	0.005

* Complete-case study

LD=left dominant; MACCE=major adverse cardiac and cerebrovascular events; MI=myocardial infarction; RD+CD=right dominant or co-dominant

**Table 5:** Independent predictors of major adverse cardiac and cerebrovascular events according to univariate and multivariate Cox analyses.

Variables	Univariate		Multivariate	
	HR (95% CI)	*P*-value	HR _adjusted_ (95% CI)	*P*-value
Left dominance	1.87. (1.21-2.88)	0.008	2.18 (1.39-3.42)	0.001
Age	1.98 (1.31-2.28)	0.04	1.01 (1.00- 1.04)	0.07
Male gender	1.31 (0.73-1.98)	0.78	1.10 (0.70-1.74)	0.66
Obesity	0.91 (0.75-1.21)	0.89	0.99 (0.96-1.02)	0.61
Vascular dissase	2.82 (1.79-4.98)	<0.001	1.75 (1.24-2.47)	<0.001
COPD	1.69 (1.21-2.61)	0.03	1.43 (1.15-2.52)	0.08
Recent MI	1.49 (0.78-4.52)	0.64	1.42 (0.81-3.14)	0.32
Severe LV dysfunction	1.72 (0.89-2.92)	0.03	1.53 (1.01-2.60)	0.02
Diabetes mellitus	1.11 (0.92-1.20)	0.72	1.10 (0.82-1.87)	0.24
Hyperlipidemia	1.02 (0.88-1.31)	0.81	0.98 (0.76-1.38)	0.82
eGFR < 60 ml/min	2.92 (1.61-5.09)	<0.001	2.28 (1.75- 3.32)	<0.001
Incomplete revascularization	1.32 (0.78-2.42)	0.09	1.21 (0.84-2.07)	0.15
SYNTAX score	1.24 (0.88-2.24)	0.11	0.98 (0.85-1.01)	0.19

CI=confidence interval; COPD=chronic obstructive pulmonary disease; eGFR=estimated glomerular filtration rate; HR=hazard ratio; LV=left ventricular; MI=myocardial infarction Omnibus test for multivariate Cox model resulted in *P*<0.001.

## DISCUSSION

The present study adds novel information on the relation between the type of coronary dominance and clinical outcomes of CABG. We found an increased risk of major adverse clinical events (all-cause death, non-fatal MI, coronary revascularization) in LD patients as compared to non-LD patients.

According to several studies, left coronary dominance has been identified as a poor prognostic factor for not only patients with ACS, but also for patients who underwent elective percutaneous coronary intervention^[3,4]^. LD patients with ACS have been shown to be hemodynamically more unstable both before and after intervention than non-LD patients^[8]^. İlia et al.^[9]^speculated that RCA related collaterals in RD patients limit the infarct area in left coronary system obstructions. On the other hand, paucity of coronary collateral circulation and unbalanced distribution of the coronary blood flow enhance the procedural risk during the temporary balloon occlusion in LD patients^[10,11]^. Although there is a negative prognostic effect of LD in the patients who underwent percutaneous intervention, such a case has not been clearly demonstrated for elective CABG patients in our study. Sudden decreasing of myocardial perfusion may be negligible with optimal myocardial protection during surgical revascularization with cardiopulmonary bypass, contrary to the high-risk percutaneous manipulation of dominant LAD or Cx in LD patients. Although there was no significant difference between the two groups in terms of early results in our dataset, there was a strong association between MACCE and LD in the long term. Development of functional coronary collaterals has been shown to be smaller in LD patients than in RD patients^[12]^. This morphological pattern may cause the LD patients to be more vulnerable to ongoing atherosclerotic process after CABG. Finally, it seems reasonable to act aggressively in risk modification and secondary protection strategies to improve long-term outcomes in LD patients after discharge.

### Limitations

This study has several limitations that are inherent to its retrospective design. Firstly, emergency or urgent cases were excluded due to lack of appropriate records as a result of rapid preoperative evaluation. Considering that LD patients with ACS are hemodynamically more unstable compared to others, it is possible that the present study may underestimate the effect of coronary dominance in daily practice, particularly in terms of early results. Although we have done a detailed analysis of the risk profiles of the patients, there are probably still unmeasured variables. Randomization does not seem feasible since coronary dominance is an anatomically non-modifiable factor, but the prospective data extraction process would be particularly useful in determining important variables, such as treatment compliance and lifestyle changes after discharge. Finally, our main interest was to investigate the prognostic value of coronary dominance in this study. Differently designed studies about the pathophysiological processes are needed to elucidate the underlying mechanisms of our findings.

## CONCLUSION

LD was an independent predictor of long-term major adverse clinical events. In patients who underwent elective CABG, the negative prognostic effect of LD is more pronounced in the long term than in the early period after the operation. Therefore, it should be considered in the outpatient follow-up to improve the long-term outcome of CABG.

**Table:** 

Author's roles & responsibilities	
ES	Substantial contributions to the conception or design of the work; or the acquisition, analysis, or interpretation of data for the work; drafting the work or revising it critically for important intellectual content; final approval of the version to be published
DC	Substantial contributions to the conception or design of the work; or the acquisition, analysis, or interpretation of data for the work; final approval of the version to be published
OB	Drafting the work or revising it critically for important intellectual content; final approval of the version to be published; final approval of the version to be published
